# Mathematical Models of Cancer Cell Plasticity

**DOI:** 10.1155/2019/2403483

**Published:** 2019-10-31

**Authors:** Hasitha N. Weerasinghe, Pamela M. Burrage, Kevin Burrage, Dan V. Nicolau

**Affiliations:** School of Mathematical Sciences, Queensland University of Technology, Brisbane 4000, Australia

## Abstract

Quantitative modelling is increasingly important in cancer research, helping to integrate myriad diverse experimental data into coherent pictures of the disease and able to discriminate between competing hypotheses or suggest specific experimental lines of enquiry and new approaches to therapy. Here, we review a diverse set of mathematical models of cancer cell plasticity (a process in which, through genetic and epigenetic changes, cancer cells survive in hostile environments and migrate to more favourable environments, respectively), tumour growth, and invasion. Quantitative models can help to elucidate the complex biological mechanisms of cancer cell plasticity. In this review, we discuss models of plasticity, tumour progression, and metastasis under three broadly conceived mathematical modelling techniques: discrete, continuum, and hybrid, each with advantages and disadvantages. An emerging theme from the predictions of many of these models is that cell escape from the tumour microenvironment (TME) is encouraged by a combination of physiological stress locally (e.g., hypoxia), external stresses (e.g., the presence of immune cells), and interactions with the extracellular matrix. We also discuss the value of mathematical modelling for understanding cancer more generally.

## 1. Introduction

Cancer is a disease involving, initially, abnormal cell growth with the potential to invade locally and—later—spread to other organs. In normal cells, the highly complex processes of cell division and death are controlled by myriad genes. Proliferation, differentiation, and apoptosis of cells are controlled by the activities of those genes and balance normal cell growth while appropriately regulating programmed cell death. Cells become cancerous when mutations accumulate in the various genes that control cell proliferation and the cell cycle, death, and responses to stress, but how these mechanisms operate and the interplay between them continues to be mysteries. Cancer cells appear to behave, in some sense, as “autonomous entities,” growing without control to form tumours. Spreading cancer cells from their primary tumour to other parts of the body—through blood and lymph,—causes cancer metastasis. Once this state has been reached, treatment options become limited, and the disease is usually fatal.

The tumour microenvironment (TME) contains different types of cells and is a key actor in the cascade of local invasion and progression towards metastasis. Apart from malignant cells, it consists of cells of the immune system, tumour vasculature and lymphatics, fibroblasts, and pericytes, *inter alia*. Interactions between these cells actually give rise to the TME (from the normal local environment preceding the lesion). Additionally, the TME comprises the extracellular matrix (ECM), which provides a physical scaffold for *in situ* tumour growth. The adhesion of cells to the ECM is key to their movement out of and into the TME. Growth factors within the TME interact with cell surface receptors and impart to local tissue its tensile strength and elasticity [1].

**Figure 1:**
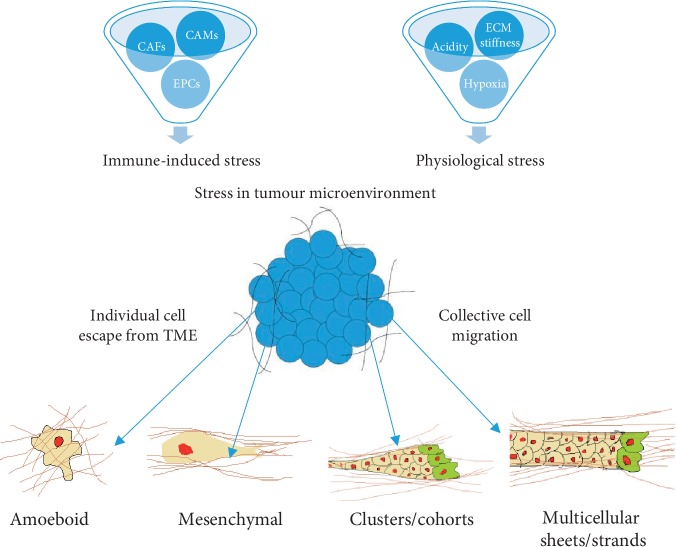
The tumour microenvironment (TME) promotes cancer cell plasticity because of the effect of ECM stiffness, acidity, hypoxia, and the presence of immune cells in TME. Cancer cells can survive in TME through epigenetic changes and also escape to more favourable environments. To escape from stress within the TME, cancer cells use individual or collective cell migration mechanisms. Two individual cell migration methods are amoeboid and mesenchymal migration. Cancer cells can migrate from the TME by collective cell migration mechanisms, in cluster or multicellular sheets.

Cell migration can be broadly classified into single cell migration and collective migration modes. These molecular programs are associated with a characteristic structure of the actin cytoskeleton, integrins, matrix-degrading enzymes, cell-cell adhesion, and signalling towards the cytoskeleton [2]. ECM stiffness, hypoxia, nutrient deprivation, acidity, and different cell populations strongly affect cell plasticity in motility [3]. The epithelial-mesenchymal transition (EMT), loss of cell-cell, and cell-ECM interactions (amongst other processes) contribute to escape mechanisms for cancer cells from the locally stressful TME. Some of the relationships between these factors and their role in promoting stress in the tumour microenvironment are illustrated in Figure 1.

In the context of the above discussion, identifying first-order principles and key biological mechanisms in cancer cell plasticity is clearly indispensable to a clear understanding of cancer progression. In particular, these principles are likely to be important in the therapeutic context of blocking or slowing the spread of cancer cells: a major challenge for developing modern cancer treatment therapies. Since biological experiments are expensive, time-consuming, ethically challenging and sometimes downright impossible with existing technology, mathematical models can provide an independent, experiment-free check of hypothesis consistency, ideally focusing (or moderating or altering) hypotheses before experimental work as well as in a feedback loop of model-experiment-model. More specifically, mathematical models can be used to describe cancer at various scales and act as an exploratory tool to complement experimental work. Furthermore, mathematical models can be used as a predictive tool. Quantitative descriptions of cancer-driven mechanisms can lead to the development of new and novel cancer treatment therapies [4]. In this review, we explore the importance of quantitative models in cancer cell plasticity and how these models can be used to design new therapy strategies and/or optimise the benefit from existing therapy options.

## 2. Mathematical Models of Cancer Cell Plasticity

The strength of a mathematical model rests in its ability to combine experimental data, consolidating it into a coherent framework, which can be used to predict the overall (or precise) dynamics of a system. Mathematical models are very useful in identifying the parameters that are most sensitive to the system and they allow for logical reasoning beyond the provision of experiments. In the specific context of tumour growth, mathematical models can, for example, quantify the links of three-dimensional tumour-tissue architecture with growth, invasion, and underlying microscale cellular and environmental characteristics. Ideally, these approaches can lead to the design of new, targeted experiments and strategies for cancer treatments [5–8].

Mathematical modelling and computer simulation allow us to explore the so-called “what-if” scenarios describing potentially complex biophysical, chemical, and physiological processes that are often beyond the reach of experimental or clinical protocols. This might be due to the protocols being expensive, invasive, hard-to-capture, or highly variable.

Despite oft-held beliefs to the contrary in the biological and medical sciences, mathematical models can in fact be simple and used to capture the “sense” of the system under study, often through phenomenological approaches. They may, of course, also be very complex and this complexity implies that the computer simulation is very intensive, takes a long time to run and/or require substantial resources of another type (e.g., computer memory).

There is also an additional issue: potentially a large number of parameters need to be calibrated against available data. If there are insufficient data, then the model may well be “undetermined”, that is, unable to discriminate between different hypotheses. In addition to the calibration issue, the models need to be validated so that they can be used in predictive settings. Validation can be performed by running computer simulations using just some of the available data and then seeing how well the outputs derived from the simulations match the data that were “held back” when calibrating.

Models may be static (representing known behaviour) at a specific point in time. Models may also evolve in time, for example, describing the action of a drug, or they may evolve in time and space, for example describing the growth and motion of a tumour. Models may also be stochastic in that they try to represent processes, such as diffusion, that are fundamentally stochastic in nature.

In outline, and broadly speaking, there are three mathematical modelling techniques having been so far used to understand cancer dynamics and plasticity: discrete, continuum, and hybrid. Discrete models track and update individual cells according to a set of biological rules [7, 9]. Continuum models consider the tumour tissue as a continuous medium, and differential equations are used for modelling [6]. Hybrid models combine the benefits of the discrete and continuous modelling and techniques, and describe chemical reactions and tissue landscapes in a single model [10, 11].

In the discussion below, we organise the individual models/studies under review along these lines, but, importantly, we also taxonomise the models by their primary findings (and aims). In particular, we distinguish between models whose aim is (a) the understanding of cell plasticity and tumour progression in their own right versus (b) those with clinical implications, that attempt to understand the effect of therapy on tumour progression and/or how to optimise treatment efficacy. We are also not aiming here to cover every existing model or to go into the details of specific models. Rather, our aim is to give the reader a flavour for each type of mathematical approach. We also aim to illustrate the kinds of insight that mathematical models of different kinds can provide with respect to understanding basic cancer cell biology but also with respect to therapy design and optimisation.

### 2.1. Discrete Cell Modelling

A discrete model can address the behaviour of one or more *individual* cells as they interact with one another and the microenvironment. In this way, discrete models are best suited to understanding cancer progression and cell plasticity-related changes at the level of the individual cell. Discrete or individual-based models can be generally further divided into two distinct categories: lattice-based and lattice-free. Lattice-based models track cells within a rigid “grid,” while off-lattice models have no such restriction [7, 9] and permit the model cells to move freely within the simulation space.

A substantial advantage of discrete models is that they operate with simple transition rules, e.g., a cell can divide and its daughter will be placed in a neighbouring cell, rather than using constitutive differential equations. This makes them fundamentally accessible to nonexperts, since the rules can be designed and understood by the nonmathematician. Rule-based approaches also are more fundamentally suited to describing biological interactions, which are themselves rule-based, involving individual agents. This is a major advantage but brings with it two drawbacks: firstly, these models generally require much more computational power (less of an issue in the era of high-performance computing than previously) and secondly, because of the absence of equations, these models lend themselves less to drawing simple conclusions about the system under study. For example, it is more difficult to find a simple relationship between a fundamental parameter, e.g., cell motility and a variable of interest, e.g., time to plasticity-induced invasion of local stroma.

#### 2.1.1. Lattice-Based Models

Lattice-based models themselves are different from one another. One way to think about this is that there are different conceptual methods can be used to implement the lattice: allowing either exactly one, more than one, or less than one cell per lattice site (in the latter case, basically sites represent subcellular compartments rather than entire cells). More specifically, there are models that use *cellular automata* (CA), *lattice gas cellular automata* (LGCA) models, and *cellular Potts* models (CP). In CA models, a single lattice site can hold a single cell, while LGCA models allow multiple cells in a single site (at the expense of some extra computational effort). CP models use multiple lattice sites to represent each cell [7]. Lattice-based models all have in common that they are inherently stochastic, since the movement of each cell is a stochastic event.

*(1) Discrete Models of Cancer Therapy Optimisation and Design*. An early stochastic cell population model was developed by Donaghey using Monte Carlo simulation in 1980 [12]. In this approach, the direction in which a cell moves is determined by generating a uniform random variable and using this to simulate random cell movement (seeing which subinterval it lies in in order to determine a direction). Although this tool was not developed specifically for studying tumours, Donaghey developed a digital simulation language (CELLSIM) which can be used to model cell population kinetics generally and used tumour growth as a case study of its use. CELLSIM was used to compare the results of models with those obtained from the actual population and to develop novel, optimised schedules for chemotherapy treatment for cancer patients. In many ways, this early work played a large part in shaping the field. It preceded later, more sophisticated models that had access to more experimental data.

During approximately the same period, Dutching and colleagues began to develop tumour growth models based on *control theory*, methods of *system analysis*, *automata theory*, and computer science. They simulated the results for different chemotherapy treatment methods to optimise the most suitable treatment as well as the treatment schedule [13–16]. Some of these mathematical models considered the tumour growth in a nutrient medium [17] and attempted to describe the spatial structure and the time behaviour of disturbed cell growth in the two-dimensional and three-dimensional cell spaces. They helped to set the stage specifically by showing the potential, in the long run, of optimising chemotherapy schedules by using simulation experiments as a powerful new tool *prior* to clinical therapy.

Kansal et al. [18] developed a three-dimensional CA model for brain tumour growth considering “Gompertzian” growth. This is a standard approach in mathematical biology generally, in which initially the growth is exponential, followed by slower growth rate until a plateau is reached as tumours grow. This model, extended by Schmitz et al. [19] to study the effects of treatments by considering three treatment parameters (sensitivity of proliferative cells, differing susceptibility of cells in the arrested state, and mutational response of the tumour to treatment), predicted the composition and dynamics of the tumour at selected time points, which was in agreement with medical literature. An important prediction of this latter model is the emergence and eventual dominance of a second tumour clone with a different genotype to the primary (indicating, amongst other things, the flexibility of this type of model). The model of Schmitz et al. incorporates several important and novel features (in its time), e.g., how to model proliferative and nonproliferative cells, an isotropic lattice, and an adaptive grid lattice.

Some of the most recent work on CA models in tumour growth involves modelling three-dimensional invasive solid tumour growth in heterogeneous microenvironments under chemotherapy [20] by modifying an earlier CA model developed by Jiao and Torquato through taking into account a variety of microscopic-scale tumour-host interactions, such as short-range mechanical interactions between tumour cells and tumour stroma, degradation of ECM by the invasive cells and oxygen/nutrient gradient-driven cell motion [21, 22]. The simulations indicated—crucially for patient care—that, in a mathematical context, constant dosing is generally more effective than periodic dosing, due to the resulting continuous high drug concentration in constant dosing. The effects of geometrically confined microenvironment and nonuniform drug dosing were also investigated in this model, with complex resulting predictions.

*(2) Discrete Models of Fundamental Tumour Plasticity, Growth, and Invasion*. Smolle and Stettner developed a lattice-based model that studies tumour growth and invasion under the influence of surrounding stroma and tumour cells for cell division, migration, and death in tumour tissues [23]. The model indicated how cellular functions and microenvironmental factors influenced histological tumour patterns. The model was later improved to study the histological patterns in melanoma [24]. Basically, these models suggest that the resulting morphological patterns closely depend on the preset functional properties of tumour cells, with autocrine and paracrine factors introducing specific modifications of the histology and, therefore, that comparisons of clinical histological samples with computer generated patterns may lead to a better “functional” interpretation of clinical samples.

Ferreira et al. [25] developed a lattice-based growth model for primary carcinomas by considering biological factors such as cell division, motility, and death controlled by the diffusion of growth factor. The results of the model indicated that the growth patterns are compact with gyration radius, surface roughness, and number of peripherical cells scaling.

Hatzikirou and Deutsch introduced a microscopic modelling method called *lattice gas cellular automata* (LGCA) to study and analyse the effects of the microenvironment on cell migration by developing two models to address the motion in an environment providing directional information and considering the influences of orientation of the cells [26]. Hatzikirou et al. [27] established a LGCA model of tumour invasion to analyse observed travelling-front behaviour in a homogeneous environment of two interacting populations of tumour cells and necrotic material. These models predicted the velocity of the travelling invasion front, which in these models depends on fluctuations arising from the motion of the discrete cells at the front of the growing tumour. The calculated width of the travelling front was found to be proportional to the velocity of the front (cells). Finally, Chopard et al. [28] developed an LGCA model to predict the velocity of the travelling invasion front, which, in their findings, strongly depends upon fluctuations that arise from the motion of the discrete cells at the front.

The *cellular Potts model* (CPM) was developed by Graner and Glazier [29] for modelling cell sorting and was applied thereafter to various phenomena in biological development. The results indicated that long-distance cell movement leads to “sorting,” with a logarithmic increase in the length scale of homogeneous clusters within the primary tumour. They found two successive phases: a rapid boundary-driven creation of a low cohesivity cell monolayer around the aggregate, followed by a slower (and boundary-independent) internal rearrangement. The basic CPM has been since extended to also account for the effects of growth factors [30], extracellular materials [31], nutrients [30, 32], or other diffusing chemicals [33].

#### 2.1.2. Lattice-Free Models

In lattice-free approaches, cells are at liberty to move in any direction and any distance consistent with the underlying biological, physical, and chemical processes. A complicating feature of these models is, of course, collision detection between the cells since, unlike in lattice-based approaches, the cells are not confined to discrete “voxels.”

*(1) Lattice-Free Models of Fundamental Tumour Plasticity, Growth, and Invasion*. Anderson and Chaplain developed a discrete mathematical model (as part of a hybrid formulation) for the formation of the capillary sprout network under the effect of tumour angiogenic factors (TAF) secreted by a solid tumour [34] based on a “discretized” form of a system of nonlinear partial differential equations (i.e., equations that evolve both in time and in space). These equations describe the initial migratory response of endothelial cells to the TAF and fibronectin. The discretized model tracks individual (model) endothelial cells at the sprout tips and incorporates anastomosis, mitosis, and branching explicitly. The results of this model are complex but importantly indicate that both chemotaxis and haptotaxis are necessary for the formation of a capillary network at a large scale. This model was later extended to describe invasion and metastasis by focusing on three key variables: tumour cells, host tissue (ECM), and matrix-degradative enzymes associated with the tumour cells [35]. The results suggested that haptotaxis is crucial for both invasion and metastasis.

Drasdo and Hohme introduced an off-lattice model for tumour growth [36]. This model assumed that each cell is an elastic, sticky particle of limited compressibility and deformability, which is capable of active migration, growth, and division. The *standard metropolis* algorithm was used to simulate a friction-dominated stochastic dynamics driven by physical interactions, which was also used by Drasdo et al. for a Monte Carlo approach to analyse the dynamics of tissue-cell populations [37]. These models make quite specific predictions about the growth of avascular tumours with a necrotic core that are approximately spherical. In particular, a key finding is that depletion of glucose or oxygen (or both) depletion seems to determine the size of the necrotic core but not the size of the tumour.

*(2) Discrete Models of Cancer Therapy Optimisation and Design*. A recent off-lattice model to integrate physical dynamics and cell signalling was proposed by Lettort et al. [38]. They presented an open-source package called PhysiBoss that combines intracellular signalling using Boolean modelling (MaBoss) [39] and multicellular behaviour using agent-based modelling (PhysiCell) [40]. These tools are useful for studying *heterogeneous* cell population responses to treatment, mutation effects, and different modes of invasion or isomorphic morphogenesis events. The models have specifically studied heterogeneous cell fate decisions in response to tumour necrosis factor (TNF) treatment and explored the effect of different treatments on the behaviour of several resistant mutants. The findings of these models are very diverse indeed and beyond the scope of this article, but the thorough review provided in [40] gives specific examples of the power of this system for studying different cancer biology and treatment problems.

### 2.2. Continuum Modelling

*Continuum models* treat tumours as a collection of tissue and the underlying processes are described by differential equations. Compared to discrete models, continuum model parameters are easier to develop and analyse as the models often represent a mean field approach compared with Monte Carlo methods. Furthermore, such models are often amenable to mathematical analysis that allows, for example, steady-state solutions to be determined analytically. Unfortunately, the trade-offs relative to discrete models is that (a) the mathematics is generally inaccessible to nonmathematicians and (b) that the resulting constitutive equations may contain simplifications of the biology (e.g., assuming continuity or heterogeneity) that may make the results highly theoretical and of limited clinical relevance.

The general form of a continuum model is a partial differential equation (PDE) that describes dynamics in time and space. Typically, we may have a function *u*(*t*, *x*) that is a function of time *t* and position *x*. Here, *u* may describe the density of a population of cells and *x* lies in some spatial domain. The governing equations take the form

(1) $$\begin{array}{c} \frac{\partial u}{\partial t}=\nabla_{x}\left(D\left(x\right)\nabla_{x}\right)+f\left(t,x,u\right). \end{array}$$

Here, ∂*u*/∂*t* means the partial derivative with respect to *t*, ∇_*x*_ is the gradient derivative with respect to *x*, *D*(*x*) is the *diffusion tensor*, and *f*(*t*, *x*, *u*) describes any chemical reactions the cells may be experiencing. If *D*(*x*) is a constant, then the spatial domain is said to be homogeneous. However, if *D* is a function of space, the problem is said to be heterogeneous. Models of this type need to be provided with an initial condition for *u* at *t* = *t*_0_, an initial time, and boundary conditions on the boundary of the domain. This then guarantees the existence of a (unique) solution to the model.

#### 2.2.1. Continuous Models of Fundamental Tumour Plasticity, Growth, and Invasion

Various mathematical models of tumour growth by diffusion have been developed in the literature, which is rich along these lines. Controlling for the mitosis of tissues, tumour growth was modelled by considering sources such as negative feedback mechanism [41], discontinuous switchlike mechanisms [42, 43], nonuniformly distributed mitotic inhibitors [44, 45], effects of a necrotic core [46], and others.

McElwain and Ponzo developed a model for the growth of solid tumours with nonuniform oxygen consumption [47], focusing on necrosis within a tumour. The results of the model were compared with the simpler model developed by Greenspan [48], and this analysis identified significant differences between the growth patterns of these two models. In some cases, for instance, the predicted radius of (just) the necrotic core is much larger in the McElwain and Ponzo model than the predicted outer radius of the whole tumour in the Greenspan model. A related model, considering apoptosis as a volume loss mechanism [49], reproduces clinically observed growth patterns and predicts that, under certain conditions, a dormant state can exist without the formation of a central coagulative necrotic region.

Chaplain and Britton presented a mathematical model for the production of a growth inhibitory factor (GIF) within a multicell spheroid representing a tumour by assuming that the GIF is produced by cells within the spheroid in some prescribed nonlinear, spatially dependent manner [50]. The results of simulations of this model suggest that using a nonlinear, spatially dependent source function that reflects the heterogeneity of the interior of multicell spheroids is sufficient to produce a GIF concentration profile within the spheroid, as observed experimentally. Chaplain extended this model to study avascular growth in solid tumours and considered two mathematical models describing different aspects of solid tumour growth and development (angiogenesis and vascular growth) for tumour angiogenesis factor (TAF), under the assumption of linear (Fickian) diffusion and endothelial population balance equation, by considering the general *conservation equation* for endothelial cell density [51]. The predictions of this work are complex but, crucially, the models suggest that there appears to be a natural critical domain size in this system, in which the size of a carcinoma is diffusion-limited and the carcinoma is an avascular state. While in this state, no invasion can take place, but, once vascularized, the models predict rapid exophytic growth.

Anderson and Chaplain described the formation of the capillary sprout network in response to TAF supplied by a solid tumour. Their model takes account of the essential endothelial cell-ECM interactions via the inclusion of the matrix macromolecule fibronectin. It consists of a system of nonlinear partial differential equations (PDEs) describing the initial migratory response of endothelial cells to the TAF and the fibronectin [34]. Anderson et al. moderated this mathematical description for tumour invasion and metastasis, describing the interactions of the tumour cells, ECM, and matrix-degradative enzymes (MDE) [35]. This last model was based on a system of reaction-diffusion-chemotaxis equation, and the authors later extended it by considering cell interactions with ECM, macromolecules (MM), MDEs, and oxygen [52–54]. In outline, the findings of this body of work are two. Firstly, tumour plasticity is driven by selective pressure in the hostile microenvironment; secondly, the loss of cell adhesion is an indispensable driver of plasticity and invasion.

Instead of the more complex PDE models described above, which directly represent physical space, tumour growth and treatment can be modelled based on ordinary differential equations (ODEs) that describe the evolution of a system in time only, such as *exponential growth*, *logistic equation, and linear-quadratic* models [55]. Clearly, these models are inherently less realistic in general, but for specific problems (in which we may not be interested in spatial properties of tumours), they are suitable because they are easier to analyse. ODE models can essentially be derived from the PDE model by saying that *u* does not depend on space. Thus, there is no diffusion term and the ODE model becomes

(2) $$\begin{array}{c} \frac{\text{d}u}{\text{d}t}=f\left(t,x,u\right),\;u\left(t_{0}\right)=u_{0}. \end{array}$$

In some cases, a time-dependent control *c*(*t*) that minimises some pay-off can be found. In this case, we can modify this ODE as described very recently by Sharp et al. [56]:

(3) $$\begin{array}{c} \frac{\text{d}u}{\text{d}t}=f\left(t,x,u,c\right). \end{array}$$

Andasari and Chaplain derived a system of ODEs for intracellular modelling of cell-matrix adhesion during cancer cell invasion by applying the law of mass action [57]. The model accounts for reactions between cell surface receptor integrins, the matrix glycoprotein fibronectin, and actin filaments in the cytoskeleton. The results suggested that rearrangement of actin filaments with integrin/fibronectin complexes near adhesion sites and interaction with fibrillar fibronectin produces the force necessary for cell migration (taking account of cell-matrix adhesion forces).

Gerich and Chaplain modified and extended the model developed by Anderson et al. [35] by developing a local and, respectively, a nonlocal model for cancer cell invasion [58]. The local model was based on haptotaxis (directional motility) and the nonlocal model was based on cell adhesion. Assuming that cancer cells proliferate according to the logistic growth law, they observed in simulations of the model that as sensing radius goes to zero, the nonlocal model converges to a related system of reaction-diffusion-taxis equations. Domschke et al. extended this nonlocal model to explore the effect of varying cell-cell and cell-ECM adhesion properties of the cancer cell for multiple cancer cell populations [59]. The computational simulation results of the model showed a range of heterogeneous invasion patterns as a consequence of several possible changing cell-cell and cell-matrix adhesion scenarios. Bitsouni and colleagues [60] developed a nonlocal mathematical model describing cancer cell invasion and movement as a result of integrin-controlled cell-cell adhesion and cell-matrix adhesion, and transforming growth factor-beta (TGF-*β*) effect on cell proliferation and adhesion, for two cancer cell populations with different levels of mutation. The model consists of partial integrodifferential equations describing the dynamics of two cancer cell populations, coupled with ODEs (so is, in some sense, a hybrid of PDE and ODE modelling) describing ECM degradation and the production and decay of integrins with a parabolic PDE governing the evolution of TGF-*β* concentration. They used this model to study aggregation and travelling wave dynamics of cancer cells [61], quantitatively characterising the effect of cancer mutation rate on the speed of cancer invasion.

Friedman and Reitich developed a model concentrating on the case where at the boundary of the tumour, the birth rate of cells exceeds their death rate [62]. A new formulation for this model and the model described in [49] was developed by Cristini and colleagues, who considered the tumour core to be nonnecrotic with no inhibitor chemical species present [63]. This model assumed that tumour genetics completely determine the morphological behaviour, and the TME is not taken into account. They observed that, for highly vascularized tumours, while they grow unbounded, their shape always stays compact and invasive fingering does not occur. Macklin and Lowengrub extended this model by considering effects of the interaction between the genetic characteristics of the tumour and the TME on the resulting tumour progression and morphology [64], concluding that the range of morphological responses can be placed into three categories, depending on the TME: (a) tissue invasion via fragmentation due to a hypoxic microenvironment, (b) fingering, invasive growth into nutrient-rich, biomechanically unresponsive tissue, and (c) compact growth into nutrient-rich, biomechanically responsive tissue. These findings corroborate experimental data that suggests the importance of the impact of microenvironment on tumour growth, morphology, and implications for cancer therapy.

#### 2.2.2. Free Boundary Models for Tumour Growth

A free boundary (FB) model is a special type of differential equation model in which we wish not only to find the solution but also where the solution is actively interacting with the spatial medium and, consequently, the domain itself is unknown. FBs arise in biological models when there is an effect from the medium, e.g., the TME affecting the tumour or an area of the spatial domain becomes active from a normal inactive state. Several models of this type have been developed, all of a highly theoretical nature, some aimed at simply proving the existence of solutions to these difficult problems.

Chaplain and Stuart [65] developed a free boundary model for the diffusion of tumour angiogenesis factor (TAF) into the surrounding host tissue, explaining local anastomosis. The model includes finite boundaries, critical distance between tumour and neighbouring vessels, natural decay, and, respectively, sink term for TAF.

Friedman and Reitich [62] studied a free boundary model for the growth of a nonnecrotic, vascularized tumour where the tumour expands due to cell proliferation or death under the level of a diffusing nutrient concentration. They observed that if the tumour doubling time is large compared to the time scale of the diffusion of the nutrient, the radius of the tumour converges to a stationary radius and the convergence is very (i.e., exponentially) fast. On the other hand, the stationary solution is generally unstable and the tumour size increases exponentially fast for a large set of possible initial data.

Bazaliy and Friedman [66] studied a free boundary problem for the tumour growth with arbitrary initial shape using an elliptic-parabolic system, establishing existence and uniqueness of solutions for small time intervals. Chen and Friedman [67] considered a system of two hyperbolic equations and two elliptic equations to model tumour growth using a free boundary problem. Hyperbolic equations are used for the densities of cells in proliferating and quiescent states, respectively, while elliptic equations are used for the concentration of nutrients and pressure. The existence, uniqueness, and regularity of the solution are proved for small time intervals. Chen et al. [68] also modelled a hyperbolic free boundary problem with proliferating and quiescent cell populations and proved that the stationary solution is linearly asymptotically stable.

Friedman [69] studied a free boundary tumour model with three population of cells—proliferating, quiescent, and necrotic—by assuming tumour tissue is a fluid subject to the Stokes equation. Sources of the Stokes equation are determined by the proliferation rate of (proliferating) cells. They proved that for the coupled system of PDEs for the densities of three types of cells, the nutrient concentration, fluid velocity, and pressure have a unique smooth solution, with a smooth free boundary for a small time interval. Friedman and Hu [70] studied aggressiveness of a tumour by considering a free boundary tumour growth modelled by the Stokes equation. In this model, they used two parameters: proliferation rate (*μ*) and cell-cell adhesiveness (*γ*) which measure the aggressiveness as a quotient,  *μ*/*γ*. As the value of this parameter increases, the model predicts that the tumour will lose its spherical shape, develop “fingers,” and become invasive.

Cui and Friedman [71] studied a free boundary problem for a nonlinear system of two ODEs. One ODE is singular at some points, including the initial point  *r*=0, and hence, the initial value problem has a one-parameter family of solutions. They proved that there exists a unique solution to this free boundary problem.

Friedman [72] introduced a free boundary multiscale tumour model including the effects of gene mutations on the population density of the tumour cells. Two time scales are tumour growing time and cycle time of individual cells. The model is formulated as a system of PDEs for population densities of cells and concentrations of oxygen and chemokines. They proved the existence and uniqueness of the solution, and properties of the free boundary are also established.

Xu et al. [73] studied a free boundary problem for tumour growth under the influence of delay, by assuming that the process of proliferation is delayed compared with apoptosis (because of the time required for mitosis). They formally prove that if the ratio of the diffusion time scale to the tumour doubling time is small, the volume of the tumour is self-limiting, i.e., it will either disappear or evolve to a dormant state. Xu and Wu [74] analysed a free boundary model of tumour growth with angiogenesis. They proved the existence and stability of the steady-state solutions when the rate at which the tumour attracts blood vessels is constant. Similarly, Zhang and Tao [75] studied the growth of an avascular tumour comprising two different cell types (proliferative and quiescent) with different chemotactic responses to extracellular nutrients using a free boundary model, proving global solvability of the model.

#### 2.2.3. Continuous Models of Cancer Therapy Optimisation and Design

Enderling et al. [76] presented a mathematical model for the growth and invasion of a solid tumour into a domain of breast tissue using a *linear-quadratic* (LQ) model. The model predicted that the single high dose of radiotherapy delivered by targeted intraoperative radiotherapy would result in eliminating sources of recurrence. On the other hand, fractionated external beam radiotherapy would, in their model, eliminate stray tumour cells but allow cells with loss of heterozygosity (LOH) in tumour suppressor genes (TSGs) or cell-cycle checkpoint genes to pass on low-dose radiation induced DNA damage and, consequently, mutations that may favour the development of a new tumour.

Based on the PDE model developed by Anderson and colleagues [35] and the model developed in [76], Enderling et al. presented a system of ODEs to mathematically study breast cancer development, local treatment, and recurrence [77]. They apply different simulated treatment strategies (surgery, adjuvant external beam radiotherapy, and targeted intraoperative radiotherapy) in the context of their model to identify different sources of local recurrence and to discuss their prevention in the clinical setting.

Byrne and Chaplain studied growth of nonnecrotic tumours on the effect of inhibitors using a model with two reaction-diffusion equations that describe the distribution of externally supplied nutrient and inhibitor species and an integrodifferential equation that governs the evolution of outer radius of the tumour [78]. They represented evolution of the tumour as a free boundary problem. Implications of the model for treatments of cancer are also discussed at a level of detail beyond the scope of the present article but essentially highlighting the complex effects of encouraging vs suppressing an immune response and vascular delivery of drugs and nutrients to tissue.

### 2.3. Hybrid Modelling

Hybrid or continuum-discrete models can bridge the gap between the cellular scale and the tumour (or even organism) scale. In the hybrid approach, tumour tissue is modelled using both discrete and continuum elements. In general (but with exceptions), oxygen, nutrient, drugs, growth factors, and certain tissue features are described as continuum fields and cells are described as discrete elements. There are sometimes subtle elements in interfacing the two approaches and to get the model to behave appropriately.

Anderson developed a hybrid mathematical model of solid tumour invasion to study how the geometry of the growing tumour is affected by mutation-driven tumour cell heterogeneity focusing on four key variables: tumour cells, the ECM, matrix-degradative enzymes, and oxygen [52]. The results predicted that local tumour cell-ECM interactions, not cell-cell interactions, control the overall geometry of the tumour. Gerlee and Anderson [79] studied the emergence of the glycolytic phenotype of clonal evolution in cancer, using a hybrid model in which the continuum part of the model is contained in the system of nonlinear PDEs modelled in [35]. They analysed the influence of tissue oxygen concentration and ECM density on the dynamics of the model output. The results suggested that the combined effect of the oxygen concentration and matrix density creates an environment in which a “glycolytic phenotype” gains a selective advantage. Anderson and colleagues also developed a hybrid model of cancer invasion considering cellular and microenvironmental factors simultaneously and interactively [54], whose outputs indicate that the genetic makeup of a cancer cell may “realize” its plastic/invasive potential through a clonal evolution process driven by TME selective forces.

Jiang et al. [30] developed a mathematical model for avascular tumour growth that spans three distinct spatial scales. At the level of the cell, a lattice Monte Carlo model describes cellular dynamics (proliferation, adhesion, and viability). At the subcellular level, a Boolean network regulates the expression of proteins controlling the cell cycle. At the extracellular level, reaction-diffusion equations describe the chemical dynamics (nutrient, waste, growth promoter, and inhibitor concentrations). The model predicted TME conditions required for tumour cell survival and ranges of the diffusion coefficients of growth promoters and inhibitors, while (using the same parameters) the model also accurately matched experimentally determined spheroid growth curves under different external nutrient supply conditions.

Jeon et al. developed an off-lattice hybrid model for tumour growth and invasion [80], building on earlier work by Anderson and Chaplain [34]. This semidiscrete model includes cell-cell and cell-ECM interaction, cell proliferation and cell death. The authors used the Langevin equation in the form of force balance to develop the model, which makes no implicit or explicit assumption about the nature of cellular movement. They found that cell-cell adhesion and haptotaxis are the key drivers of tumour growth and morphology.

## 3. Perspectives

**Figure 2:**
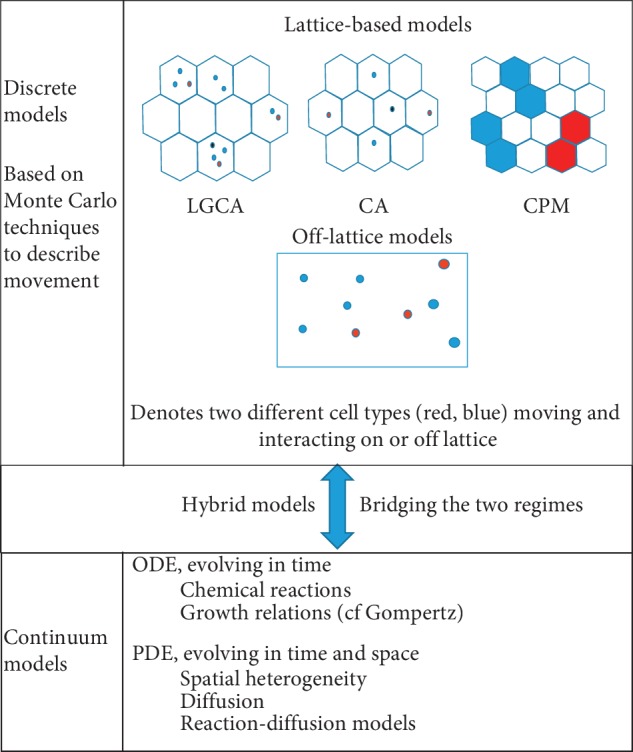
D*iscrete cell* modelling, *continuum* modelling, and *hybrid* modelling, respectively, can be used to mathematically study tumour progression and metastasis. Discrete models can be classified into lattice-based models and lattice-free models. Lattice-based models can be further classified as Lattice Gas Cellular Automata (LGCA), Cellular Automata (CA), and Cellular Potts models (CPMs). Stochastic models and finite-difference approximation methods are used in lattice-free approaches. For continuum modelling, ordinary differential equations (ODEs) and partial differential equations (PDEs) are used. Logistic power and the Gompertz law are the basic ODEs used for continuum modelling. PDEs such as reaction-diffusion and partial integro-differential equations are also used for continuum modelling. Finally, hybrid models combine discrete and continuum approaches by modelling cell dynamics as *discrete* and certain tissue features such as oxygen, nutrient, drugs, etc. as *continuum fields*. To develop these mathematical models, various tumour microenvironmental factors such as matrix-degrading enzymes, extracellular matrix (ECM), oxygen, growth factors, inhibitors, etc. are considered.

We have here reviewed a large number of fairly diverse quantitative models of cancer cell plasticity, falling roughly into three categories: discrete, continuum, and hybrid, each with strengths and weaknesses relative to a specific research question. The schematic relationships of these classes of models to one another are illustrated in Figure 2. For the convenience of the reader, we have also summarised (nonexhaustively) the principal findings of these modelling studies and the mathematical techniques used in each case in Table 1.

We now compare, in brief, the strengths and weaknesses of the different types of models reviewed here. One may consider different approaches when one is addressing roughly different degrees of detail. For example, the discrete lattice-free models perhaps include the most detailed information about the relevant biology, but are also the most computationally expensive, restricting their applications to relatively smaller scales or requiring the use of supercomputers. In contrast, discrete lattice-bound models coarse-grain the free space into discrete lattice sites, reducing the computational cost regarding cell movements, often at the cost of losing spatial precision, which is a good compromise depending on the questions being asked. The continuum models further coarse-grain discrete cells into a continuous density, with the aim of enabling such models to simulate large-scale phenomena but, again, at the cost of losing all local spatial information, e.g., local cell-cell interactions. If this loss impairs the modelling approach from answering important questions but we still require the simulation of large-scale phenomena, we may then consider using hybrid modelling.

We now offer some perspectives on the future development of this exciting and highly active area of research. An important question is how such models can be used in a clinical context. In a so-called “personalized medicine” clinical setting, there may be a need to have results available from computer simulations very quickly, to aid, say, invasive operations. Recently, there has been a move to run such simulations on cheap graphical processing units (GPUs) that can be deployed in standard PCs that may speed up simulations by several orders of magnitude, placing this technology at the bedside. The simulations can be combined with advanced visualization techniques.

Models can also be used to enhance either missing data or data that hard to collect. Statistical or machine learning/artificial intelligence techniques can then be used to characterize or learn about important features. This is not possible if data are sparse, since these approaches require large amounts of high-quality data for training the system.

Models can also be used to make sense of data that are highly variable, for example data collected from a cohort of patients in some disease state. This can be done by calibrating not a single model to, say, the mean of the highly variable data but by calibrating a population of models (with the same framework but different parameters) against the data. This population of models can be studied statistically, quantifying the uncertainty, or run forward in time to provide probabilistic outcomes, that is beyond just a single model. Such an ensemble is sometimes called a *virtual population* [81, 82].

Before data collection, a new experimental protocol requires testing and optimisation. To identify what types of data should needed for the experiment, a mathematical model can be used. Therefore, mathematical models represent a natural framework for experimental protocols. To improve the quality and the practical use of mathematical models and to generate qualitative and quantitative predictions, stronger collaboration of mathematicians and biomedical researchers is needed.

The models of tumour cell plasticity we surveyed here illustrate and colour the discussion above. For example, using a discrete model, one can identify biological factors such as heterogeneity and interaction between cells. However, it can be difficult to investigate the model behaviour because many simulation runs are required. Continuum models provide tools to describe movement and aggregation patterns of cell populations under various tumour microenviornmental factors. In this way, for example, the effect of microenviornmental factors for the tumour progression and the mechanism of cancer cell plasticity can be observed. Hybrid models combine discrete and continuum descriptions of cancer biology by bridging the gap between the cellular scale and tumour scales. Hybrid models attempt to combine the best features of discrete models and continuum models.

The challenge, going forward, for mathematical modelling in cancer in general and plasticity specifically, is moving from a descriptive to a prescriptive power, for example, generalizing their findings after appropriate validation so that they can be used to design *a priori* novel effective treatment strategies, rather than showing a good match with existing experimental or clinical data, as tends to be the case.

## 4. Conclusions

Mathematical models can be used to study the different stages of tumour progression such as avascular and vascular tumour growth, angiogenesis, invasion, and metastasis under the effects of TME factors. Because of tumour cell plasticity, cancer cells are able to escape from the TME and, further down the cascade, resistance to therapies as well as immune system evasion arises. By studying quantitative models, the factors and influences leading to cell plasticity can be identified. For example, as many models now confirm, ECM stiffness, hypoxia, nutrient deprivation, hypoxia, and acidity all promote cell plasticity and motility within the TME [2]. Most quantitative models described above are developed considering cell-cell adhesion, cell-ECM adhesion, hypoxia, etc. Escaping from the TME as a collection of cells or as an individual cell, together with patterns of cell aggregation, can also be better understood with these types of models.

## Figures and Tables

**Table 1:** The important models reviewed here are organised by modelling approach and a summary of their key finding(s). The list is nonexhaustive.

Reference	Model	Submodel	Key result
Donaghey [12]	Discrete	CA	Higher proportion of cells will enter the *G*_0_ absorbing cell state as the total cell population gets larger.
Düchting and Vogelsaenger [13]	Discrete	CA	After treatment, undamaged *G*_0_ cells are recruited into the cell cycle again and stimulate tumour growth.
Duchting and Dehl [14]	Discrete	CA	Critical initial number of tumour cells of a tumour nucleus is necessary for the growth of a tumour. Additional high-influence variables are the mean life span of a tumour cell and the amount of tumour cell loss.
Smolle and Stettner [23]	Discrete	CA	Histological tumour patterns depend complexly on the autocrine and paracrine factors.
Smolle et al. [24]	Discrete	CA	Relative degree of motility to proliferation decreases from benign to primary malignant and metastatic, but the absolute degree of motility is increasing.
Ferreira et al. [25]	Discrete	CA	Growth patterns of the tumour are compact with gyration radius, surface roughness, and number of peripheral cells.
Schmitz et al. [19]	Discrete	CA	A tumorous subpopulation is most highly favoured when the interfacial area among strains is maximized. Total volumetric fraction of nonlocalized strains is not important in tumour development.
Jiao and Torquato [21]	Discrete	CA	Quantitative properties of the host microenvironment can significantly affect tumour morphology and growth dynamics.
Jiao and Torquato [22]	Discrete	CA	Strong cell-cell adhesion can suppress the invasive behaviour of the tumours growing in soft microenvironments; cancer malignancy can be significantly enhanced by harsh microenvironmental conditions, such as exposure to high pressure levels.
Xie et al. [20]	Hybrid	CA, diffusion reaction	In chemotherapy, constant dosing is generally more effective in suppressing primary tumour growth than periodic dosing, due to the resulting continuous high drug concentration.
Hatzikirou et al. [27]	Discrete	LGCA	Width of the travelling front is proportional to the front speed.
Chopard et al. [28]	Discrete	LGCA	There is a positive effect of fibre track on glioma growth.
Graner and Glazier [29]	Discrete	CPM	Long-distance cell movement leads to sorting with a logarithmic increase in the length scale of homogeneous cell clusters.
Jiang et al. [30]	Discrete	CPM	The microenvironmental conditions required for tumour cell survival and growth promoters and inhibitors have diffusion coefficients in the range 10^−6^ to 10^−6^*cm*^2^/*h*.
Turner and Sherratt [31]	Discrete	CPM	Increased proliferation rate results in an increased depth of invasion into the extracellular matrix.
Shirinifard et al. [32]	Discrete	CPM	Simulated avascular tumours form cylinders following the blood vessels, leading to a differential distribution of hypoxic cells within the tumour.
Anderson and Chaplain [34]	Continuum and discrete (discrete model is the discretized form of the continuum model)	Diffusion-reaction equation	
Random walk model	Both chemotaxis and haptotaxis are necessary for the formation of a capillary network at large scales.		
Anderson et al. [35]	Continuum and discrete (discrete model is the discretized form of the continuum model)	Diffusion-reaction equation	
Random walk model	ECM structures can aid or hinder the migration of individual cells that have the potential to metastasis. As time increases, small cell clusters can be observed.		
Chaplain and Stuart [65]	Continuum	PDE	Possible explanation for anastomosis
Bazaliy and Friedman [66]	Continuum	PDE	Establish the existence and uniqueness of a solution for some time interval
Friedman [69]	Continuum	PDE	For the densities of three types of cells: proliferating, quiescent and necrotic, the nutrient concentration, fluid velocity, and pressure have a unique smooth solution, with a smooth free boundary for a small time interval
Chen et al. [68]	Continuum	Partial integro-differential equations	Stationary solution of the model is linearly asymptotically stable
Cui and Friedman [71]	Continuum	ODE	Initial value problem has a one-parameter family of solutions and there exists a unique solution to the free boundary problem.
Zhang and Tao [75]	Continuum	PDE	Prove the global solvability of the model
Xu and Wu [74]	Continuum	PDE	Prove the existence and stability of the steady-state solutions when the rate at which the tumour attracts blood vessels is constant.
